# A novel plasma p-tau181 assay as a specific biomarker of tau pathology in Alzheimer's disease

**DOI:** 10.1186/s40035-024-00439-4

**Published:** 2024-09-03

**Authors:** Kenji Tagai, Harutsugu Tatebe, Sayo Matsuura, Zhang Hong, Naomi Kokubo, Kiwamu Matsuoka, Hironobu Endo, Asaka Oyama, Kosei Hirata, Hitoshi Shinotoh, Yuko Kataoka, Hideki Matsumoto, Masaki Oya, Shin Kurose, Keisuke Takahata, Masanori Ichihashi, Manabu Kubota, Chie Seki, Hitoshi Shimada, Yuhei Takado, Kazunori Kawamura, Ming-Rong Zhang, Yoshiyuki Soeda, Akihiko Takashima, Makoto Higuchi, Takahiko Tokuda

**Affiliations:** 1 Advanced Neuroimaging Center, Institute for Quantum Medical Science, National Institutes for Quantum Science and Technology (QST), 4-9-1 Anagawa, Inage-ku, Chiba-shi, Chiba, 263-8555 Japan; 2 Department of Psychiatry, The Jikei University of Medicine, Tokyo, 105-8461 Japan; 3 Department of Oral and Maxillofacial Radiology, Tokyo Dental College, Tokyo, 101-0061 Japan; 4 Department of Psychiatry, Keio University School of Medicine, Tokyo, 160-0016 Japan; 5 Department of Functional Neurology and Neurosurgery, Center for Integrated Human Brain Science, Brain Research Institute, Niigata University, Niigata, 951-8585 Japan; 6 Laboratory for Alzheimer’s Disease, Department of Life Science, Faculty of Science, Gakushuin University, Tokyo, 171-8588 Japan

## Main text

Alzheimer's disease (AD), the most prevalent form of dementia, is characterized by deposits of two abnormal proteins, namely amyloid-β (Aβ) and tau, in the brain. There is growing evidence for the clinical significance of plasma phosphorylated tau (p-tau) assays in detecting AD pathology [1, 2], similar to CSF and positron emission tomography (PET) biomarkers. Currently available immunoassays for plasma p-tau detect C-terminally truncated p-tau containing the N-terminus to the mid-domain (N-p-tau) [2]. Plasma p-tau levels quantified using these N-p-tau assays, such as p-tau181, p-tau217, and p-tau231, can accurately differentiate between AD pathology and other tauopathies with high diagnostic accuracy [1, 2] and help predict future cognitive decline from prodromal phases [3]. However, one of the significant problems in the clinical use of N-p-tau assays is their inability to be a surrogate marker for tau burden in the brain given that the measurements obtained had a more pronounced association with amyloid PET than with tau PET [4]. Therefore, the need for developing blood-based tau biomarkers strongly correlated with PET-detectable tau accumulations in the brain without being affected by amyloid accumulation remains unmet.

The present study aimed to develop a novel plasma p-tau biomarker which could accurately reflect the AD-type tau burden in the brain captured by tau PET without being affected by amyloid accumulation, and could thus be interchangeable with tau PET imaging. We developed a novel mid-region-directed p-tau181 assay to detect both N- and C-terminally truncated p-tau181 fragments (mid-p-tau181), and validated its usefulness. The plasma mid-p-tau181 assay exhibited high analytical performance (Additional file 1: Supplementary Materials and Methods, Fig. S1–S5; Additional file 2: Tables S1–S5). The demographics of the study participants (*n* = 164) is shown in Additional file 2: Table S6.

**Fig. 1:**
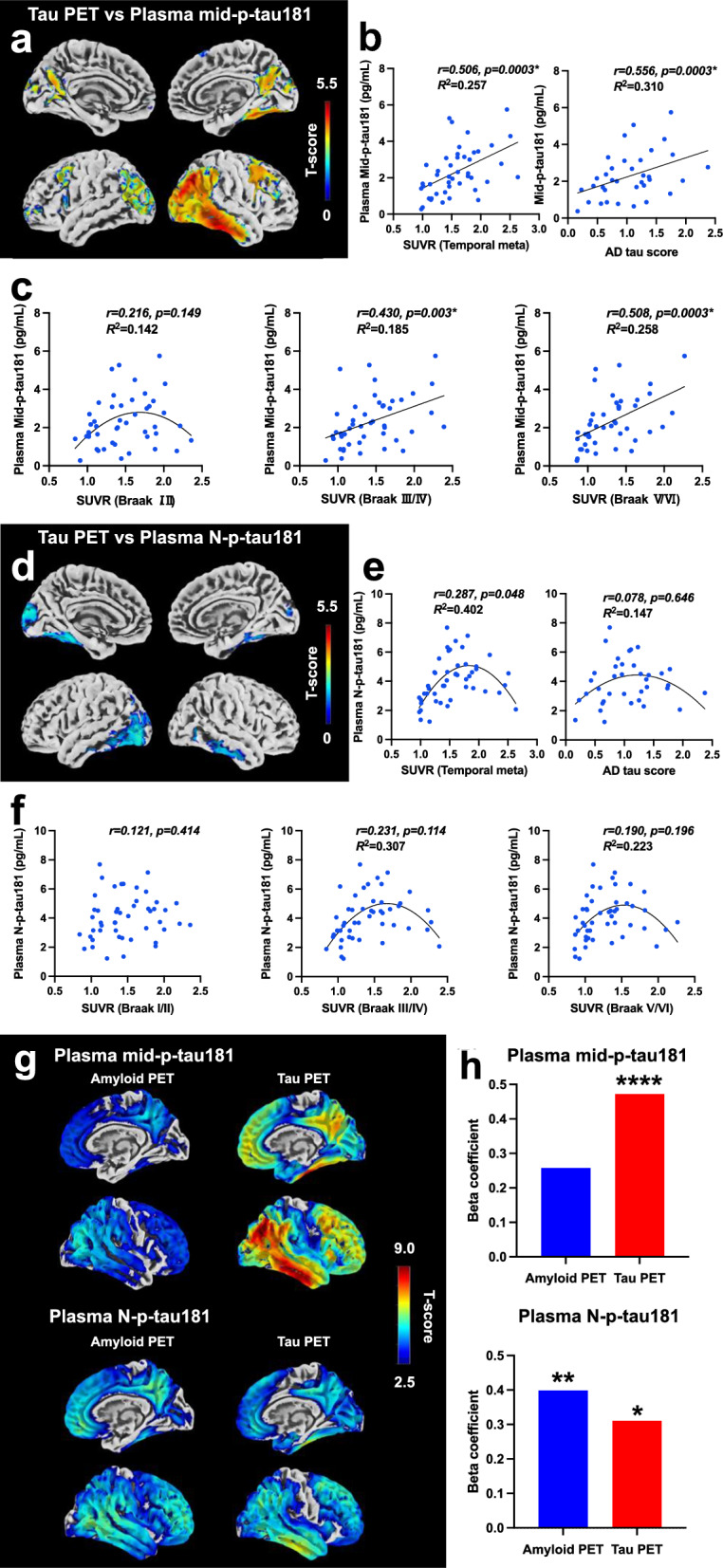
Correlations of plasma mid-p-tau or N-p-tau level with tau or amyloid PET in the subjects with AD continuum. **a** The correlation between plasma mid-p-tau181 level and ^18^F-florzorotau tau PET is depicted through its topographical representation (*P* < 0.05, FWE-corrected). **b**, **c** Scatter plots of the correlation between plasma mid-p-tau181 level and tau PET tracer accumulation in each ROI. Pearson's correlation analysis was employed to calculate the *r* and *P* values. Statistical significance was established at* P* < 0.0125, corrected for multiple comparisons using the Bonferroni method. Regression analysis, indicated by a straight or curved line, depicts the preferred model, with its goodness of fit quantified using the *R*^2^ value. **d** Topographical representation of the correlation between plasma N-p-tau181 level and tau PET (*P* < 0.05, uncorrected). **e**, **f** Scatter plots of the correlation between plasma N-p-tau181 level and tau PET tracer accumulation in each ROI. The procedure for the correlation analysis was the same as that in **b** and **c**. **g** Topographical representation of the correlations of each plasma p-tau181 assay with amyloid- (left column) and tau- (right column) PET in the CN and AD continuum cohort (*P* < 0.05, FWE-corrected). **h** Bar graphs illustrating beta coefficient values of amyloid and tau PET in multiple liner regression analysis with each p-tau assay. *P < 0.05, **P < 0.005, ****P < 0.0005. *P*-values were corrected for multiple comparisons using Bonferonni correction with the number of explanatory variables

We found a significant linear correlation between the plasma mid-p-tau181 level and the retention of tau PET tracer (^18^F-florzorotau aka PM-PBB3/APN-1607) in patients with AD continuum (Fig. 1a–c). Voxel-wise analyses revealed a positive correlation between plasma mid-p-tau181 level and tracer binding in the temporal and parietal cortices (Fig. 1a; *P* < 0.05, corrected for family-wise error [FWE]). This finding was corroborated by three different tau region-of-interest (ROI) imaging analyses: (1) the temporal meta-ROI [5], (2) the AD tau score [6], and (3) tau imaging-based Braak staging [5] (Additional file 1: Fig. S6, see Supplementary Methods for detailed procedures). The plasma mid-p-tau181 level showed significant linear correlations with the temporal meta-ROI standardized uptake value ratio (SUVR) (Fig. 1b, *r* = 0.506; *P* = 0.0003), AD tau score (Fig. 1b, *r* = 0.556; *P* = 0.0003), and SUVRs in the Braak stages III/IV (Fig. 1c, *r* = 0.403; *P* = 0.003) and V/VI (Fig. 1c, *r* = 0.508; *P* = 0.0003) but not Braak stage I/II (*r* = 0.216; *P* = 0.149) ROIs. These findings clearly demonstrate that the plasma mid-p-tau181 level could accurately reflect AD-related tau accumulation that spreads from the entorhinal cortex to the inferior temporal lobe and then to the parieto-occipital regions of the neocortex, but not tau accumulation in the Braak I/II region. Conversely, the conventional N-p-tau181 assay (Simoa pTau-181 Advantage V2.1 kit, Quanterix, MA) showed no significant linear correlations with tau PET tracer retention (Fig. 1d–f). Voxel-based analysis revealed no significant correlation between the plasma and imaging parameters in the temporal cortex upon correction for multiple comparisons (*P* < 0.05, FWE-corrected). Moreover, none of the three ROI-based analyses showed significant linear correlations with N-p-tau181 level (*P* > 0.05 after Bonferroni’s correction). Instead, inverse U-shaped nonlinear correlations were shown (Fig. 1e, f). The list of the Akaike Information Criterion (AIC) values used for selecting the fitting model for each region can be found in Additional file 2: Table S7.

Next, plasma p-tau181 levels determined by either the pre-established (N-p-tau181) or newly developed (mid-p-tau181) assay were assessed for association with ^11^C-PiB amyloid-PET and ^18^F-florzorotau tau-PET measures of pathology (Fig. 1g, h) in cognitively normal (CN) and AD continuum. Results showed that the plasma mid-p-tau181 level was more strongly associated with tau PET accumulation than with amyloid, while the plasma N-p-tau181 level was more strongly associated with amyloid PET accumulation than with tau (Fig. 1g). Multiple linear regression analysis also revealed that the plasma mid-p-tau181 level was significantly influenced only by tau PET accumulation (Amyloid PET: *P* = 0.056; Tau PET: *P* < 0.0001, adjusted *R*^2^ = 0.450). Meanwhile, the plasma N-p-tau181 level was influenced by both amyloid and tau PET accumulation (Amyloid PET: *P* = 0.002; Tau PET: *P* = 0.020, adjusted *R*^2^ = 0.420) (Fig. 1h, Additional file 2: Table S8).

Next, we conducted receiver operating characteristic (ROC) curve analyses to determine the cutoff level of each plasma p-tau181 assay for discriminating between positive and negative amyloid/tau PET. The PET finding classification was based on the cutoff values of Centiloid, imaging-based Braak staging, temporal meta-ROI SUVR, and AD tau score (Supplementary Methods). The cutoff values for the temporal meta-ROI SUVR and AD tau score were determined using ROC curve analyses in the current and previous studies [6], respectively (Additional file 2: Table S9). Results showed that both p-tau concentrations were higher in amyloid-positive or tau-positive cases compared to negative cases (Additional file 1: Fig. S7; *P* < 0.0001). Furthermore, the area under the ROC curve values for each p-tau181 assay differentiating between amyloid/tau PET positivity and negativity all exceeded approximately 0.85 (Additional file 2: Table S10). The cut-off value of N-p-tau for amyloid positivity was 2.20, while those for tau positivity ranged from 2.20 to 3.19, showing overlap between amyloid and tau discrimination. In contrast, the cut-off value of mid-p-tau for amyloid positivity was 0.749, while those for tau positivity ranged from 1.62 to 1.76, with no overlap between pathology. Furthermore, mid-p-tau181 showed substantially higher specificity for tau-PET positivity (average 89.7%) compared to amyloid-PET positivity (60.5%). Meanwhile, N-p-tau181 exhibited almost equivalent specificities for amyloid- and tau-PET positivity (76.7% vs average 83.5%, Additional file 2: Table S10). These results indicate that N-p-tau is influenced by both amyloid and tau, rising relatively early in the disease course, whereas mid-p-tau is influenced only by tau and rises at a later stage (Additional file 1: Fig. S8). Consequently, mid-p-tau181 proves to be a more specific biomarker for tau pathology, demonstrating robust discriminative power with reference to PET-based classifications.

The results of current study demonstrate that plasma mid-p-tau181 could accurately reflect the AD-type tau burden in the brain captured by florzolotau without being affected by amyloid accumulation, and could thus have a high correlation with tau PET imaging as a blood-based biomarker for detecting and staging PET-detectable AD tau pathologies in the brain.

Most current approaches for quantifying soluble p-tau via immunoassay predominantly target tau forms phosphorylated at positions 181, 217, or 231 and bearing N-terminal epitopes [2]. Various non-clinical and clinical studies have suggested that an increase in those p-tau species in biofluids could be strongly associated with Aβ aggregation [2, 4]. Therefore, concentrations of N-terminally intact p-tau measured using conventional assays have been recognized as not a genuine tau marker that reflects PET-visible tau aggregations [4]. A previous study demonstrated that all plasma p-tau species measured by N-p-tau assays were more tightly associated with amyloid PET than tau PET [4]. Conversely, our mid-p-tau181 assay strongly correlated with tau but not amyloid PET indices in AD brains. A detailed consideration of the differences between our mid-p-tau181 assay and conventional N-p-tau assays is provided in Additional file 1: Fig. S9, S10, and Discussion. The differences in performance between these assays might be influenced by the fragmentation of tau associated with tangle evolution. N-terminal and C-terminal cleavages occur in tau proteins insolubilized and deposited in the brain, which has been postulated to play a role in tangle evolution [7]. Conventional N-p-tau assays might be incapable of assessing N-terminally truncated tau species that appears with tangle maturation [7], whereas our mid-p-tau assay may be less affected by such truncation (Additional file 1: Fig. S5). Since ^18^F-florzolotau exerts high sensitivity and specificity for tau versus amyloid aggregates in the AD continuum [8], the plasma mid-p-tau181 measurement offers the blood-based proper “T” biomarker in the ATN framework, corresponding to the AD-type tau accumulation in tau PET scans. Moreover, the characteristics of mid-p-tau181 suggest its usefulness as a biomarker for predicting the therapeutic response to current disease-modifying therapies targeting Aβ. The plasma mid-p-tau181 could discriminate between tau-positive and -negative subjects with specificity greater than 85% and had a significant linear correlation with tau PET tracer retention in patients in the AD continuum. Therefore, plasma mid-p-tau181 analysis could identify patients with low-to-moderate tau burden who are more likely to benefit from Aβ-targeting therapies [9].

A limitation of the present study was that the sample size was modest, and longitudinal data were lacking. As such, clinical cohort studies assessing neuroimaging- and fluid-based biomarkers with a larger scale are required to justify the present findings and are underway.

The present study has demonstrated the capability of the newly developed plasma mid-p-tau181 assay for pursuing the dynamics of tau fragments in association with the tangle formation and for the biological staging of AD. Although tau fragments in the CSF have been measured and shown to correlate with tau pathologies [10], there are still unmet needs for implementing comprehensively validated blood-based biomarkers that accurately reflect cerebral tau burden with minimal interference by amyloid deposition. Our novel mid-p-tau assay potentially offers a biomarker with high accessibility and functionality for evaluating the evolution of neurodegenerative tau pathogenesis that has been investigated only by PET. Notwithstanding the necessity for additional proofs, this technology will also be applicable to the selection of patients most suitable for anti-Aβ therapies and the examination of efficacies of tau-targeting therapies.

## Supplementary Information

**Additional file 1: Materials and methods. Discussion. Fig. S1**. A schematic illustration tau protein showing the epitope location of the antibodies used in this study. **Fig. S2**. Standard curve of the mid-p-tau181 immunoassay. **Fig. S3**. Dilution linearity. **Fig. S4**. Spike recovery and parallelism. **Fig. S5**. Comparison of the AEB signals between our mid-p-tau181 assay and the commercially available p-tau181 assay. **Fig. S6**. List of ROIs applied to the tau PET images. **Fig. S7**. Scatter plots of the mid-p-tau/ N-p-tau 181 showing its ability to discriminate between amyloid/tau PET status determined by semiquantitative approaches in the cognitively normal and AD continuum subjects. **Fig. S8**. Trajectories of the imaging and plasma A/T/N biomarkers along with the decline in the MMSE scores. **Fig. S9**. Correlations between plasma p-tau levels assessed by each assay and amyloid/tau PET in the subjects with CN and AD continuum. **Fig. S10**. Correlations between plasma mid-p-tau and N-p-tau levels in the subjects with AD continuum.

**Additional file 2: Table S1**. Intra-assay precision (*n* = 20). **Table S2**. Inter-assay precision for quality control samples (*n* = 25). **Table S3**. Recovery rate (%Recovery) for each plasma sample in the spike recovery tests. **Table S4. **An additional study of intra-assay reproducibility (%CV) by using the data on duplicated measurement of internal quality controls when the levels of plasma mid-p-tau181 were measured in the participants. **Table S5. **An additional study of inter-assay reproducibility (%CV) by using the data on repeated measurement of internal quality controls when the levels of plasma mid-p-tau181 were measured in the participants. **Table S6.** Demographic and blood biomarker data of the participants. **Table S7.** Differences in AIC values for regression analyses of p-tau levels and tau PET parameters. **Table S8.** Multiple linear regression analysis of p-tau levels with amyloid and tau PET metrics. **Table S9.** Amyloid/Tau PET status of cognitively normal individuals and AD continuum patients assessed by semi-quantitative approaches. **Table S10.** Performance of mid-p-tau 181and N-p-tau181assays in discriminating amyloid/tau PET status in the CN and AD continuum subjects.

## Data Availability

The data supporting this study's findings are available from the corresponding author on reasonable request. Sharing and reuse of data require the expressed written permission of the authors, as well as clearance from the Institutional Review Boards as the data should be used under license from our institution (National Institutes for Quantum Science and Technology, Chiba, Japan) so are not publicly available.

## Abbreviations

AD: Alzheimer's disease
Aβ: Amyloid-β
PET: Positron emission tomography
ROI: Region of interest
SUVR: Standardized uptake value ratios
ROC: Receiver operating characteristic
