# Supplementary material for: A novel plasma p-tau181 assay as a specific biomarker of tau pathology in Alzheimer's disease
Source: Transl Neurodegener. 2024 Sep 3;13:44. doi: 10.1186/s40035-024-00439-4 (PMC11375937; doi:10.1186/s40035-024-00439-4)
Supplement: Supplementary file 1 — Additional file 1: Materials and methods. Discussion. Fig. S1. A schematic illustration tau protein showing the epitope location of the antibodies used in this study. Fig. S2. Standard curve of the mid-p-tau181 immunoassay. Fig. S3. Dilution linearity. Fig. S4. Spike recovery and parallelism. Fig. S5. Comparison of the AEB signals between our mid-p-tau181 assay and the commercially available p-tau181 assay. Fig. S6. List of ROIs applied to the tau PET images. Fig. S7. Scatter plots of the mid-p-tau/ N-p-tau 181 showing its ability to discriminate between amyloid/tau PET status determined by semiquantitative approaches in the cognitively normal and AD continuum subjects. Fig. S8. Trajectories of the imaging and plasma A/T/N biomarkers along with the decline in the MMSE scores. Fig. S9. Correlations between plasma p-tau levels assessed by each assay and amyloid/tau PET in the subjects with CN and AD continuum. Fig. S10. Correlations between plasma mid-p-tau and N-p-tau levels in the subjects with AD continuum. [file 40035_2024_439_MOESM1_ESM.pdf]

Additional file 1

Materials and methods

Participants

We recruited a cohort of 186 individuals comprising 43 Cognitively normal (CN) individuals, 30 patients with mild cognitive impairment (MCI) due to AD, 31 patients with dementia due to AD, 52 patients with progressive supranuclear palsy (PSP), and 30 patients with other frontotemporal lobar degeneration (FTLD) syndromes between January 2018 and September 2022. The other FTLDs comprised 12 cases with corticobasal syndrome, 10 cases with behavioral-variant frontotemporal dementia, 7 cases with frontotemporal dementia and parkinsonism linked to chromosome 17 MAPT, and 1 case of primary progressive aphasia. CN individuals were those aged older than 40 years who had no history of neurological and psychiatric disorders, had a Mini-Mental State Examination (MMSE) score of ≥28, or had a Montreal Cognitive Assessment score of ≥26 and Geriatric Depression Scale score of ≤5 [1]. Patients with MCI and dementia due to AD underwent clinical evaluations. Cognitive impairment severity was defined as MMSE < 24 and Clinical Dementia Rating (CDR) ≥ 1 for dementia and MMSE ≥ 26 and CDR = 0.5 for MCI [1]. Patients with PSP and other FTLD syndromes were diagnosed according to established criteria previously reported [1,2].

In the present study, we selected cases for further analysis using strict criteria based not only on clinical diagnosis but also on amyloid and tau PET, since the accumulation of the tau PET ligand, ^{18}F-florzorotau could reflect both AD and non-AD tau. the diagnosis of patients with MCI and dementia due to AD required brain amyloid positivity in PET scans, and they were combined and categorized into the AD group based on the concept

of the AD continuum. We excluded patients with PSP and other FTLD who had positive amyloid PET results to eliminate the influence of mixed pathology. Those with other FTLDs were heterogeneous and categorized into the FTLD group. Furthermore, CN individuals with a positive result on either or both amyloid and tau PET scans were also excluded as they were considered to have been in the preclinical AD stage. Amyloid positivity was defined based on ^{11}C-Pittsburgh Compound B (PiB)-PET visual inspection performed by a minimum of three specialists with expertise in the field [2]. Tau PET negativity in CN individuals was defined according to the AD tau score (< 0.1986) and PSP tau score (< 0.3431) as reported previously [1]. These scores, which were calculated from ^{18}F-florzorotau PET images via our established machine learning algorithm, possess a high degree of sensitivity and specificity in discriminating CN individuals from patients with AD and PSP [1]. Consequently, after excluding 3 CN individuals and 11 MCI, 2 AD, 2 PSP, and 4 FTLD patients according to the aforementioned criteria, our final cohort consisted of 164 participants comprising 40 CN individuals and 48 AD, 50 PSP, and 26 FTLD patients.

Blood sampling

We obtained blood samples through venous puncture on the same day as the PET scan. A total of 8 mL of blood was collected in ethylenediaminetetraacetic acid-containing tubes. After collection, plasma was separated by centrifugation for 10 min at 2000*g*, aliquoted into polypropylene tubes, and then stored at −80°C until analysis.

Measurement of blood biomarkers

We developed a novel immunoassay able to quantify plasma levels of both N- and C-

terminally truncated p-tau181 fragments run on a highly sensitive automated digital ELISA (Enzyme-Linked Immuno Sorbent Assay) platform (Simoa HD-X Analyzer, Quanterix, Lexington, KY, USA) and measured the levels of p-tau181 including such fragments in human plasma. The details of the procedures for method validation of this original immunoassay are described below. We also quantified plasma levels of the A/T/N biomarkers [3] (Aβ42, Aβ40, p-tau181, and neurofilament light chain (NfL) instead of total tau in the original paper) utilizing the Simoa platform (Quanterix) equipped with validated assay kits. Procedures were performed following the manufacturer's instructions. This study employed the Aβ42/Aβ40 ratio as a proxy for cerebral amyloid burden. Plasma p-tau181 measured using the commercial kit (Simoa pTau-181 V2.1 Assay, Quanterix) was defined as N-p-tau181, whereas plasma p-tau181 measured using the originally developed immunoassay run on the Simoa system was defined as mid-p-tau181. All plasma samples were diluted four times with the respective sample diluent before the assays to minimize matrix effects. All plasma samples were run in duplicate with the same lot of standards. The relative concentration estimates of plasma biomarkers were calculated according to their respective standard curves.

A new assay for both N- and C-terminally truncated p-tau181 (mid-p-tau181)

As shown in Supplementary Fig. S1, an anti-tau mouse monoclonal antibody against a mid-portion of tau protein ranging from 151 to 163 amino acid residues was coupled to paramagnetic beads (Quanterix) and used for the capture antibody. As a detector, we used the AT270 mouse monoclonal antibody (Invitrogen) specific for the threonine 181 phosphorylation site. The detection antibody was conjugated to biotin following the manufacturer’s instructions (Quanterix). We used the human p-tau181 standard in the Tau

(Phospho) [pT181] Human ELISA Kit (Invitrogen) as the calibrator for our mid-p-tau181 assay. All plasma samples were diluted four times with the Tau Calibrator Diluent (Quanterix) prior to the assays to minimize matrix effects. The assay procedure followed that for the pTau-181 Advantage V2.1 Kit, except for changes to the capture antibody used as mentioned above. The details of the assay procedures have been described previously [4, 5]. All plasma samples were run in duplicate with the same lot of standards. Fluorescent signals were converted to average enzyme per bead (AEB) numbers as described previously [6] and then concentrations of plasma mid-p-tau181 were extrapolated from four-parametric logistic curves of AEBs generated with known calibrator concentrations.

Plasma mid-p-tau181 levels were measured using the previously described assay on the Simoa HD-X Analyzer (Quanterix) at the National Institutes for Quantum Science and Technology. To ensure that measurements were performed in a blinded manner, unique identifiers that did not contain any participant-related information were attached to the blood sample tubes sent from the clinical department to the investigator who measured the plasma mid-p-tau levels. The samples were decoded only after data sharing with the participating clinical scientists had been completed. Internal quality control samples were analyzed in duplicates at the start and end of each run to determine within- and between-run variations.

Validation of the newly developed mid-p-tau181 assay

1. Standard curve for the mid-p-tau181 immunoassay

Supplementary Fig. S2 shows the standard curve for our novel mid-p-tau181 assay, demonstrating that mid-p-tau181 was detected with high sensitivity. The curve was

generated by analyzing the p-tau181 standard in the Tau (Phospho) [pT181] Human ELISA Kit (Invitrogen) in duplicate measures and fitting the digital signals on a four-parameter logistic curve. The goodness of fit was 0.9999.

2. Limit of detection (LOD) and lower limit of quantification (LLOQ)

We prepared 16 aliquots of blank sample (Tau Calibrator Diluent) and measured "background" signals of our novel plasma mid-p-tau181 assay on the Simoa HD-X Analyzer (Quanterix). Signals measured on the Simoa Analyzer were quantified by a common unit, namely average number of enzymes labels per bead (AEB). Thereafter, the LOD of the assay was determined as an interpolated mid-p-tau181 concentration derived from the mean plus 2.5 SD value of AEBs for the blank samples. The LOD of the assay, which requires 50 μL of plasma, was 0.1144 pg/mL.

The LLOQ of the assay was determined as an interpolated p-tau concentration derived from the mean plus 10 SD value of AEBs for blank samples. The LLOQ of the assay was 0.3770 pg/mL.

3. Intra-assay precision

Twenty samples with different concentrations (0.039, 6.25, 25, and 100 pg/mL) of recombinant p-tau181 were prepared for analysis of intra-assay precision, and measured AEBs in one experiment. Intra-assay precision was determined by calculating within-run coefficient of variation (CV) for those samples.

Intra-assay precision was robust with CVs between 1.1% and 3.5 % (Table S1).

4. Inter-assay precision for quality controls and repeatability of the standard curve

We prepared three recombinant p-tau samples and two plasma samples with different plasma mid-p-tau181 concentrations for quality control experiments and measured the mid-p-tau181 levels in those samples five times on different days. Inter-assay precision

was determined by calculating the CV of the AEB signals between the runs for those samples.

The %CV of the AEBs at different concentrations were 5.56, 5.09, 9.19, 5.30, and 5.96 %, respectively (Table S2). These results indicated that our novel mid-p-tau181 assay showed good inter-assay precision (< 10%).

5. Dilution linearity

To explore dilution linearity, a plasma sample with a moderate mid-p-tau181 concentration was used, and twofold serial dilutions (×2, ×4, ×8, ×16, and ×32) of the sample were generated using the sample diluent until the theoretical concentration reached the LLOQ. Serially diluted samples were analyzed in duplicate.

The dilution linearity experiments demonstrated that an examined sample can be diluted to a concentration just below the LLOQ and still provide a reliable quantification after the serial dilution (Fig. S3).

6. Spike recovery and parallelism

For the spike recovery tests, four aliquots of each two plasma samples with different mid-p-tau181 concentrations were prepared and spiked with 0, 0.15, 0.3, and 0.6 pg of mid-p-tau181 in 360 μL of solution (containing patient plasma, sample buffer, and spiked peptide). These eight (4 × 2) aliquots were analyzed in quadruplicate on the same run. To evaluate parallelism, two spike recovery curves were made, starting from the non-spiked solution to the 0.6 pg-spiked solution. Recovery rates (%Recovery) were calculated by subtracting the endogenous mid-pTau181 concentration from the measured concentration [7].

In the spike recovery and parallelism experiments (Fig. S4), the recovery rate (%Recovery) of each sample was 84.4%–119.3% (Table S3). The parallelism of two spike

recovery curves starting from the non-spiked solution to the 0.6 pg-spiked solution is also shown in Fig. S4, with our data demonstrating that plasma samples spiked with 0, 0.15, 0.3, and 0.6 pg of mid-p-tau181 protein provided reliable recovery rates and parallelism.

7. Comparison of AEB signals for N- and C-terminally truncated p-tau181 between the established p-tau181 assay and the new mid-p-tau181 assay developed herein

To confirm the ability of our new mid-p-tau assay to detect both N- and C-terminally truncated p-tau proteins, we compared our assay with the widely used and commercially available p-tau181 assay (Simoa™ pTau-181 Advantage V2 Kit, Quanterix). We prepared both N- and C-terminally truncated p-tau proteins as described in the following sections.

7-1. cDNA construction

The cDNA of tau (aa100−251) was generated from NΔR cDNA in PCI-neo according to the modified instructions of the PrimeSTAR® Mutagenesis Basal Kit (Takara Bio Inc., R046) [8]. Briefly, 2.5 ng of NΔR cDNA was mixed in 1× PrimeSTAR® Max DNA Polymerase (Takara Bio Inc., R045) containing 0.2 μM of forward primer (5′-CACCATGGGAACCACAGCTGAAGAAGCAGGC-3′) and 0.2 μM of reverse primer (5′-GTGGTTCCCATGGTGGCGAATTCTCGAGGCTAG-3′). PCR was performed on a C1000™ Thermal Cycler (BIO-RAD) with the following steps: an initial denaturation at 98°C for 2 min; 18 three-step cycles involving denaturation at 98°C for 10 s, annealing at 55°C for 15 s, and extension at 68°C for 90 s; and a final extension at 68°C for 10 min. A PCR product was incubated with Dpn I (20 units) (New England Biolabs, R0176) at 37°C for 20 min to digest the template NΔR plasmid. cDNA of 100−251 tau was transformed into DH5α (TOYOBO, DNA-903F). After the DH5α was cultured in LB medium containing 10 mg/mL of tryptone (Gibco, 211705), 5 mg/mL of yeast extract (Gibco, 212750), 10 mg/mL of sodium chloride (Nacalai Tesque, 31320-05), and 100

μg/mL of ampicillin (Nacalai Tesque, 02739-32), the cDNA was purified by NucleoSpin® Plasmid Transfection-grade (Takara Bio Inc., 740490). The sequences of the cDNA were confirmed through DNA sequencing (FASMAC).

7-2. Cell culture

COS-7 cells at around 90% confluence were detached from 10-cm dishes (CORNING, 353003) by trypsinization (Nacalai Tesque, 32777-15) and counted. The cells were plated at 2.0×10^5 in a 6-well plate (CORNING, 3506). cDNAs (2.5 μg) were transfected with Lipofectamine 3000 according to the manufacturer's instruction (Invitrogen, L3000) 2 days after culturing. PCI-neo (Promega) was used as the empty vector. Transfection reagents were removed by a medium change 1 day after the transfection.

7-3. Preparation of cell homogenate

The cells were scraped in a buffer (1 × TBS [Nacalai Tesque, 35438-81], 1 mM EDTA [Nacalai Tesque, 15112-22]) containing protease inhibitors (5 μg/mL of pepstatin A [Nacalai Tesque, 26436-52, 5 μg/mL of leupeptin [Nacalai Tesque, 103476-89-7], 2 μg/mL of aprotinin [Nacalai Tesque, 03346-84], and phosphatase inhibitors [Nacalai Tesque, 07575-51]) and homogenized for 30 s on the crashed ice using a polytron homogenizer (NITI-ON, NS-310E). After homogenization, solutions were centrifuged (23000 rpm, 15 min, 4°C) using a TLA55 Rotor (Beckman Coulter). The supernatants were applied to the Bradford assay (Nacalai Tesque, 11617-71) to determine the total protein concentration and then stored at −80 °C.

7-4. Comparison between our mid-p-tau181 assay and the commercially available p-tau181 assay

Serially diluted cell homogenates, prepared as previously described, containing both N- and C-terminally truncated p-tau181 were applied to our mid-p-tau181 assay (Fig. S5, a

solid line) and a commercially available p-tau181 assay (Simoa™ pTau-181 Advantage V2 Kit, Quanterix). Fluorescent signals converted to AEB values obtained from both assays are plotted in the Fig. S5 (solid line: mid-p-tau assay; broken line: commercially available p-tau181 assay). The results from both assays demonstrated that AEB values were obtained in our mid-p-tau181 assay but not in the commercially available p-tau181 assay, indicating that our mid-p-tau assay can quantify both N- and C-terminally truncated p-tau181 that could not be detected with the conventional p-tau181 assay.

PET and magnetic resonance imaging (MRI) data acquisition

Amyloid and tau deposits in the brains of all participants were assessed using PET with ^{11}C-PiB and ^{18}F-florzorotau as described in other clinical trials (UMIN-CRT; number 000026385, 000026490, 000029608, 000030248, and 000043458). One PSP patient who had already been confirmed to be Aβ-negative at another facility no longer underwent ^{11}C-PiB-PET at our center. The scan protocol was described as follows: parametric ^{11}C-PiB-PET images were acquired 50–70 min after injection (injected dose: 528.5 ± 65.5 MBq, molar activity 90.2 ± 26.2 GBq/μmol); ^{118}F-florzorotau PET images were obtained 90–110 min after injection (injected dose: 186.6 ± 7.4 MBq, molar activity 244.2 ± 86.7 GBq/μmol). PET was primarily conducted using a Biograph mCT flow system (Siemens Healthcare), with some cases using the Discovery MI (GE Healthcare) (9 ^{11}C-PiB scans in CN individuals; 22 ^{11}C-PiB scans and 3 ^{18}F-florzorotau scans in AD patients; 7 ^{11}C-PiB scans in PSP patients; and 4 ^{11}C-PiB scans in FTLD patients) and an ECAT EXACT HR+ scanner (CTI PET Systems, Inc.) (three ^{11}C-PiB scans in CN individuals, four ^{11}C-PiB scans in AD patients, nine ^{11}C-PiB scans in PSP patients, and three ^{11}C-PiB scans in FTLD patients). Acquired PET images were reconstructed using the filtered back

projection method with a Hanning filter. MRI examination was conducted simultaneously with PET using a 3-T scanner (MAGNETOM Verio; Siemens Healthcare). The anatomical images were acquired using a three-dimensional T1-weighted gradient echo sequence that produced a gapless series of thin sagittal sections (TE = 1.95 ms, TR = 2300 ms, TI = 900 ms, flip angle = 9°, acquisition matrix = 512 × 512 × 176, voxel size = 1 × 0.488 × 0.488 mm^3).

Imaging analyses

All images were preprocessed using PMOD software (version 4.3, PMOD Technologies Ltd), FreeSurfer 6.0 (http://surfer.nmr.mgh.harvard.edu/), MATLAB (The Mathworks, Natick, MA, USA), and Statistical Parametric Mapping software (SPM12, Wellcome Department of Cognitive Neurology). PET images were co-registered with individual anatomical T1-weighted MR images, and standardized uptake value ratios (SUVR) images were generated using each reference region. The cerebellar cortex was the reference region for the ^{11}C-PiB-PET images. For ^{18}F-florzorotau PET images, an optimized reference region was set through an in-house MATLAB script that considered the distribution of diverse tau lesions throughout the entire gray matter and extracted optimized reference regions on an individual basis [9]. Each PET and MR image was also normalized to the Montreal Neurologic Institute space using the Diffeomorphic Anatomical Registration Through Exponentiated Lie Algebra (DARTEL) algorithm and was smoothed with a Gaussian kernel at 8-mm full-width at half maximum in voxel-wise analyses.

We performed an ROI (region of interest) analysis targeting AD pathologies on each imaging modality to quantify the regional amyloid/tau burden and cortical thinning. The

amyloid burden was assessed using a Centiloid atlas (frontal, temporal, parietal, precuneus, anterior striatum, and insula) implemented in the PMOD Neuro Tool (PMOD Technologies Ltd). Each Centiloid SUVR was calculated and converted to a Centiloid score [10] using PET data from 12 young CN individuals aged 23–43 years and 25 cases of AD patients scanned at our institution. Tau burden was assessed using ROIs targeting tau pathology associated with AD labeling through FreeSurfer, Braak staging ROIs (I/II, III/IV, V/VI) [11], and temporal meta-ROI (entorhinal, amygdala, parahippocampal, fusiform, inferior temporal, and middle temporal) [12]. We excluded the hippocampus from the Braak stage I/II ROI because of potential spill-in from the choroid plexus [2]. Additionally, we also estimated the AD tau score to assess AD-type tau burden in the brain, which was calculated using an Elastic Net model trained on tau PET data as previously reported [1]. A qualitative analysis based on the values obtained from these ROI analyses was also conducted to evaluate the presence of tau lesions. For Braak staging, the SUVR values were converted to z-values based on another young CN cohort, and the highest stage was assigned based on the average regional Z-score (>2.5). Those with stages 0–I/II were classified as tau-negative, whereas those with stages III/IV–V/VI were classified as tau-positive. The cutoff value of temporal meta-ROI SUVR was set at 1.105 to maximize the differentiation between CN individuals and AD patients during receiver operating characteristic (ROC) analysis (see Supplementary Material for detailed information). and the cutoff value of AD tau score was set at 0.1986 as described elsewhere [1]. Cortical thickness was measured using the cortical signature of AD through FreeSurfer (medial temporal, inferior temporal, temporal pole, angular, superior frontal, superior parietal, supramarginal, precuneus, and middle frontal) [13].

Statistical analyses

Statistical analyses were conducted using GraphPad Prism version 9 (GraphPad Software) and R version 4.3.1. Initially, group comparisons were performed using the Kruskal–Wallis test or Mann–Whitney U test for demographic data and measured blood biomarker values and Fisher's exact test for gender ($p < 0.05$, corrected by Dunn's multiple comparisons). Subsequently, correlation analyses were conducted to verify the association between each p-tau181 assay and each imaging biomarker. During voxel-based analyses, a linear regression model was applied using SPM12. The extent threshold was established based on the expected voxels per cluster. For multiple voxel comparisons, family-wise error corrections at the cluster level were applied [$p < 0.05$, corrected for family-wise error (FWE)]. During ROI-based analyses, Pearson's correlation analyses were performed ($p < 0.05$, corrected by Bonferroni multiple comparisons), and nonlinear regression analysis (quadratic) was conducted when no significant correlation was observed. Results were adopted when the nonlinear analysis based on Akaike's Information Criterion (AIC) showed a better fit than the linear one. Besides, we also incorporated a multiple linear regression analysis to explore the relationships between amyloid, tau, and each plasma pTau level. In addition, ability of each blood biomarker to discriminate between the presence or absence of AD pathology, as defined by amyloid or tau PET positivity, was also evaluated by calculating area under the ROC curve (AUC) values from ROC curve analyses. The Youden index maximizing sensitivity plus specificity minus one determined the optimized cutoff value. Finally, to explore the trajectories from CN to AD for each blood/imaging biomarker, we converted each biomarker value to a z-value based on CN data. Thereafter, we examined their relationship with cognitive dysfunction (MMSE score). A linear or sigmoidal 4 parameter logistic

regression analysis was adapted, and the better-fitting model was selected based on AIC.

Ethics approval and consent

This study was approved by the National Institutes for Quantum Science and Technology Certified Review Board. Written informed consent was obtained from all participants and spouses or close family members when participants were cognitively impaired. This study was registered with the UMIN Clinical Trial Registry (UMIN-CRT; number 000041383).

Discussion

Additional consideration of the differences between our mid-p-tau181 assay and conventional N-p-tau assays

Previous publications also report a correlation of N-terminal p-tau181 with tau PET [14,15], and the Lumipulse plasma p-tau 181 assay targets a similar region as our mid-p-tau181assay, shows a correlation with amyloid PET [16]. However, these analyses used the data from the cohorts including both CN and AD subjects. Our study, if we analyze the group combining the CN and AD subjects, supported those previous findings (Fig. S9); however, multiple regression analyses revealed that tau PET accumulation was the only factor correlated with mid-p-tau181 concentration (Fig. 1g and h).

A direct comparison between the measured concentrations of N-p-tau and mid-p-tau showed a linear correlation in the group combining the CN and AD subjects, but a nonlinear correlation was observed in the AD group alone similar to tau PET (Fig. S10). It has been reported that N-terminal and C-terminal cleavages occur in tau proteins insolubilized and deposited in AD brains [17]. Our results thus suggest that in the early stage of AD both the N-p-tau and the mid-p-tau assays could similarly detect the N-terminally intact p-tau fragments, but only the mid-p-tau assay could detect both N- and C-terminally truncated p-tau fragments that would appear as the disease progresses. Additionally, an increase in mid-p-tau was also observed in PSP cases (Table S4), indicating the need for comparisons with existing other fluid-based T biomarkers, such as MTBR-tau and NTA-tau [18,19]. Furthermore, including a validation cohort would be very advantageous to confirm that novel mid-p-tau181 is a genuine blood-based "T" biomarker.

References

1. Endo H, Tagai K, Ono M, Ikoma Y, Oyama A, Matsuoka K et al (2022) A Machine Learning-Based Approach to Discrimination of Tauopathies Using [18 F]PM-PBB3 PET Images. Mov Disord 37:2236–2246.

2. Tagai K, Ono M, Kubota M, Kitamura S, Takahata K, Seki C et al (2021) High-Contrast In Vivo Imaging of Tau Pathologies in Alzheimer’s and Non-Alzheimer’s Disease Tauopathies. Neuron 109:42-58.e8.

3. Jack CR Jr, Bennett DA, Blennow K, Carrillo MC, Dunn B, Haeberlein SB et al (2018) NIA-AA Research Framework: Toward a biological definition of Alzheimer’s disease. Alzheimers Dement 14:535–562.

4. Satoh-Asahara N, Yamakage H, Tanaka M, Kawasaki T, Matsuura S, Tatebe H, et al (2022) Soluble TREM2 and Alzheimer-related biomarker trajectories in the blood of patients with diabetes based on their cognitive status. Diabetes Res Clin Pract 193:110121.

5. Shinomoto M, Kasai T, Tatebe H, Kitani-Morii F, Ohmichi T, Fujino Y, et al (2021) Cerebral spinal fluid biomarker profiles in CNS infection associated with HSV and VZV mimic patterns in Alzheimer’s disease. Transl Neurodegener 10:2.

6. Tatebe H, Kasai T, Ohmichi T, Kishi Y, Kakeya T, Waragai M, et al (2017) Quantification of plasma phosphorylated tau to use as a biomarker for brain Alzheimer pathology: pilot case-control studies including patients with Alzheimer’s disease and down syndrome. Mol Neurodegener 12:63.

7. Andreasson U, Perret-Liaudet A, van Waalwijk van Doorn LJC, Blennow K, Chiasserini D, Engelborghs S, et al (2015) A practical guide to immunoassay method validation. Front Neurol 6:179.

8. Takashima A, Murayama M, Murayama O, Kohno T, Honda T, Yasutake K, et al (1998) Presenilin 1 associates with glycogen synthase kinase-3beta and its substrate tau. Proc Natl Acad Sci U S A 95:9637–9641.

9. Tagai K, Ikoma Y, Endo H, Debnath OB, Seki C, Matsuoka K et al (2022) An optimized reference tissue method for quantification of tau protein depositions in diverse neurodegenerative disorders by PET with 18F-PM-PBB3 (18F-APN-1607). Neuroimage 264:119763.

10. Klunk WE, Koeppe RA, Price JC, Benzinger TL, Devous MD Sr, Jagust WJ et al (2015) The Centiloid Project: Standardizing quantitative amyloid plaque estimation by PET. Alzheimers Dement 11: 1-15. e1-4.

11. Schöll M, Lockhart SN, Schonhaut DR, O'Neil JP, Janabi M, Ossenkoppele R et al (2016) PET Imaging of Tau Deposition in the Aging Human Brain. Neuron 89:971–982.

12. Ossenkoppele R, Rabinovici GD, Smith R, Cho H, Schöll M, Strandberg O et al (2018) Discriminative Accuracy of [18F]flortaucipir Positron Emission Tomography for Alzheimer Disease vs Other Neurodegenerative Disorders. JAMA 320:1151–1162.

13. Dickerson BC, Bakkour A, Salat DH, Feczko E, Pacheco J, Greve DN et al (2008) The Cortical Signature of Alzheimer's Disease: Regionally Specific Cortical

Thinning Relates to Symptom Severity in Very Mild to Mild AD Dementia and is Detectable in Asymptomatic Amyloid-Positive Individuals. Cereb Cortex 19:497–510.

14. Ossenkoppele R, Reimand J, Smith R, Leuzy A, Strandberg O, Palmqvist S, et al. Tau PET correlates with different Alzheimer's disease-related features compared to CSF and plasma p-tau biomarkers. EMBO Mol Med. 2021;13:e14398.

15. Karikari TK, Pascoal TA, Ashton NJ, Janelidze S, Benedet AL, Rodriguez JL, et al. Blood phosphorylated tau 181 as a biomarker for Alzheimer's disease: a diagnostic performance and prediction modelling study using data from four prospective cohorts. Lancet Neurol. 2020;19:422–33.

16. Silva-Spínola A, Leitão MJ, Nadal A, Le Bastard N, Santana I, Baldeiras I. Exploring the potential of fully automated LUMIPULSE G plasma assays for detecting Alzheimer's disease pathology. Alzheimers Res Ther. 2024;16:51.

17. Guillozet-Bongaarts AL, Garcia-Sierra F, Reynolds MR, Horowitz PM, Fu Y, Wang T, et al. Tau truncation during neurofibrillary tangle evolution in Alzheimer's disease. Neurobiol Aging. 2005;26:1015–22.

18. Horie K, Salvadó G, Barthélemy NR, Janelidze S, Li Y, He Y, et al. CSF MTBR-tau243 is a specific biomarker of tau tangle pathology in Alzheimer's disease. Nat Med. 2023;29:1954–63.

19. Lantero-Rodriguez J, Salvadó G, Snellman A, Montoliu-Gaya L, Brum WS, Benedet AL, et al. Plasma N-terminal containing tau fragments (NTA-tau): a biomarker of tau

deposition in Alzheimer’s Disease. Mol Neurodegener. 2024;19:19.

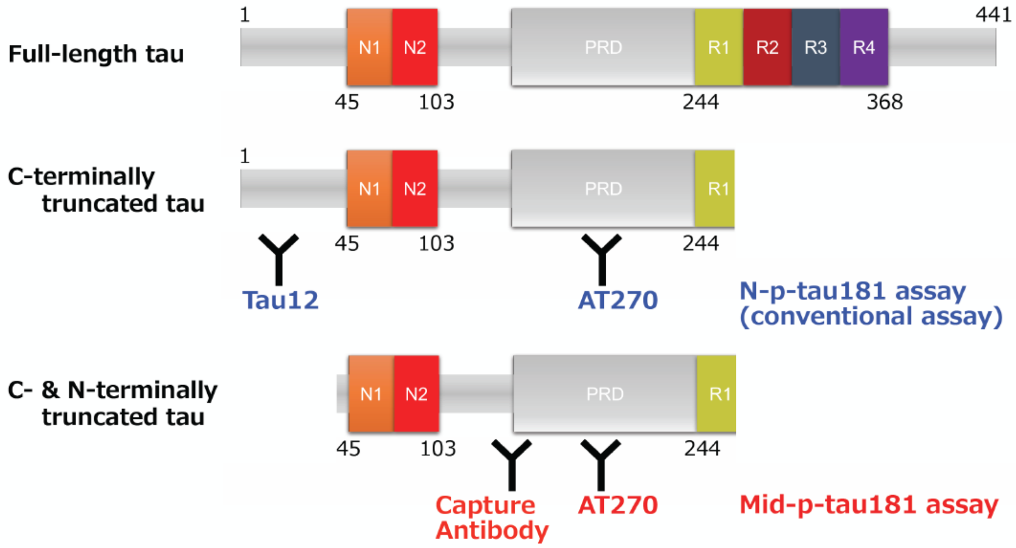

Fig. S1 A schematic illustration tau protein showing the epitope location of the antibodies used in this study.

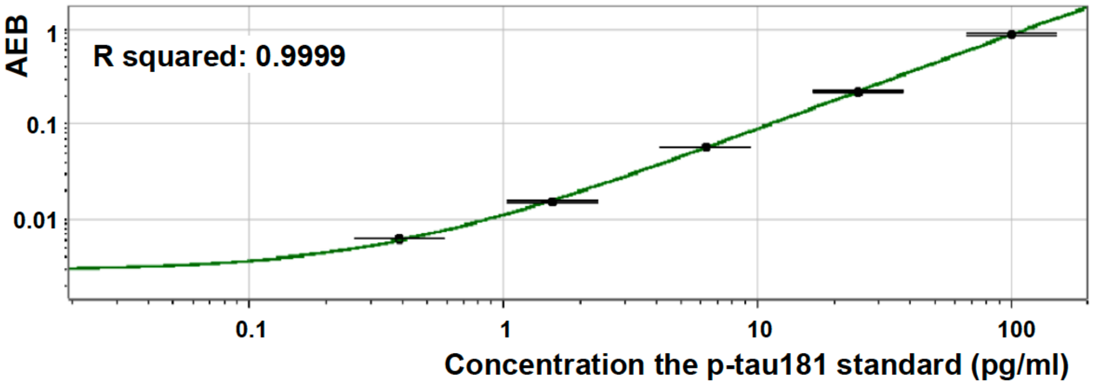

Fig. S2 Standard curve of the mid-p-tau181 immunoassay

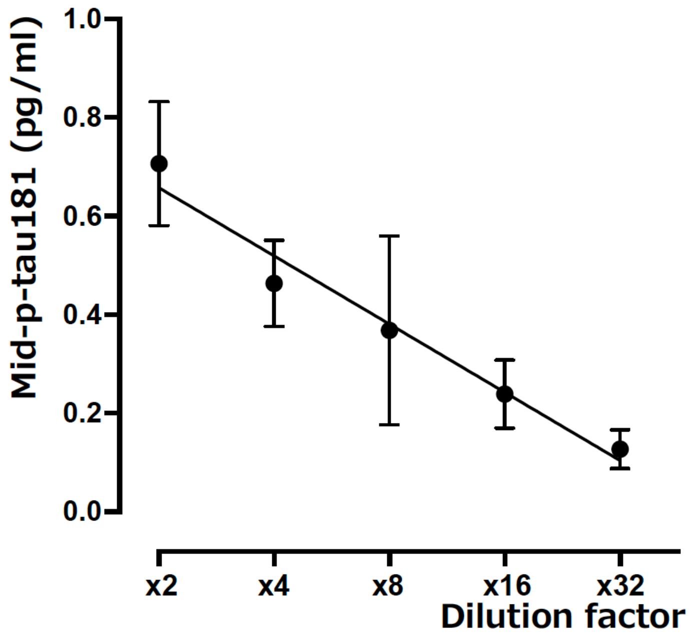

Fig. S3 Dilution linearity.

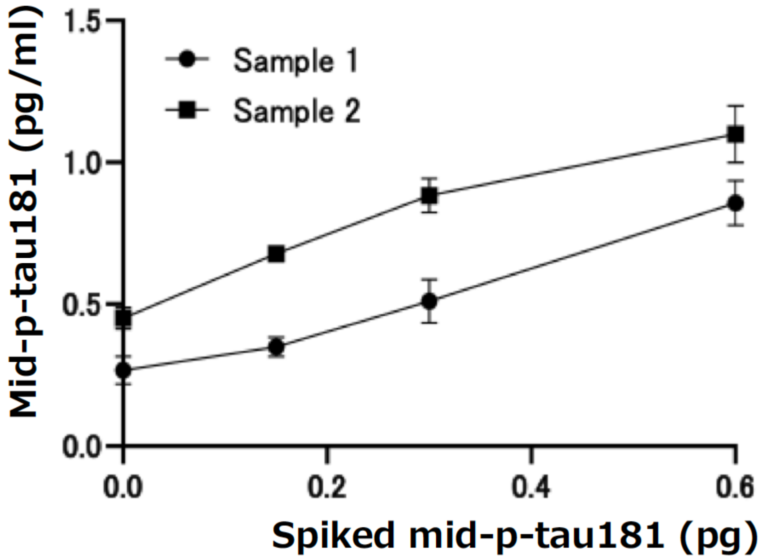

Fig. S4 Spike recovery and parallelism.

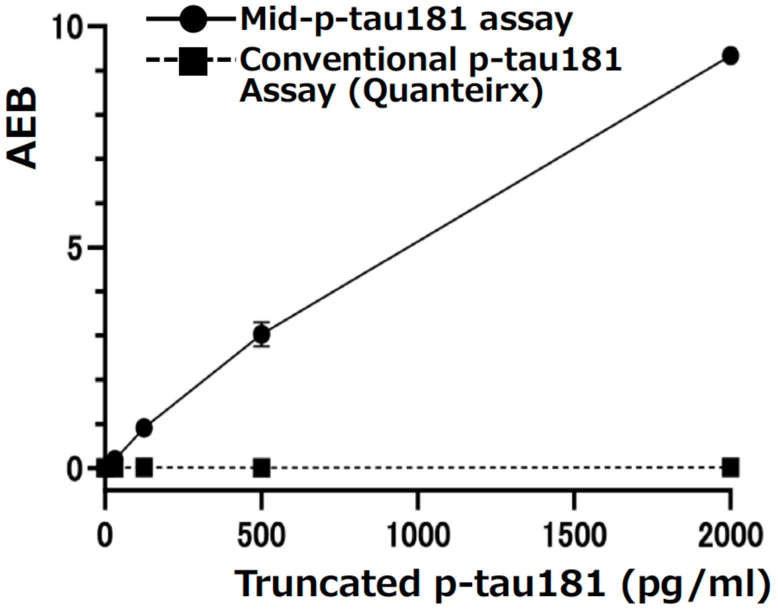

Fig. S5 Comparison of the AEB signals between our mid-p-tau181 assay and the commercially available p-tau181 assay.

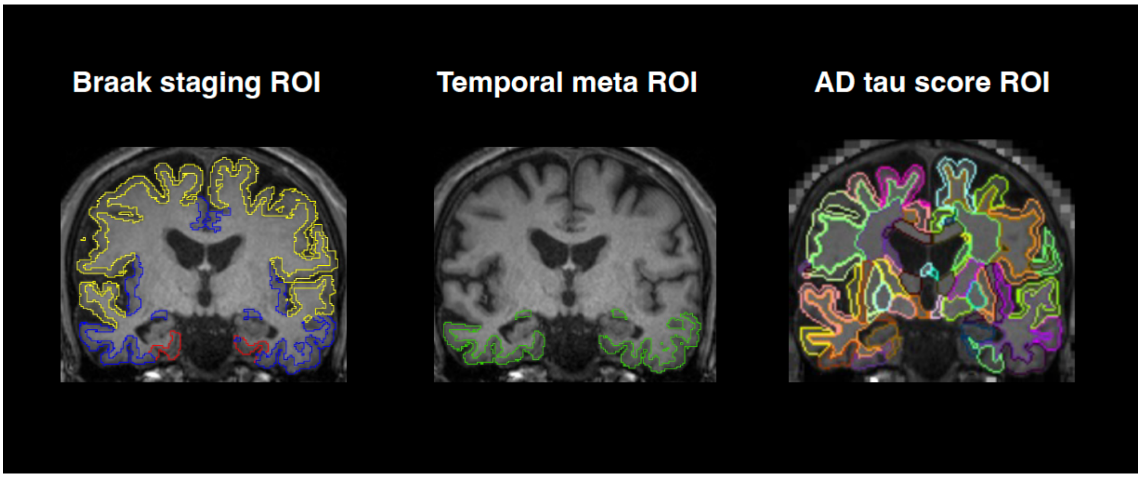

Fig. S6 List of ROIs applied to the tau PET images. From left to right, overlaid ROIs on native T1 space of representative subject: Braak staging ROI, temporal meta-ROI, and AD tau score ROI. The Braak staging ROI is color-coded, with Braak I/II, III/IV, and V/VI regions shown in red, yellow, and blue, respectively. The temporal meta-ROI is labeled in green. The AD tau score ROI represents 114 areas segmented using Mvision. Scores were derived by assigning weights to diagnostically relevant areas within these ROIs.

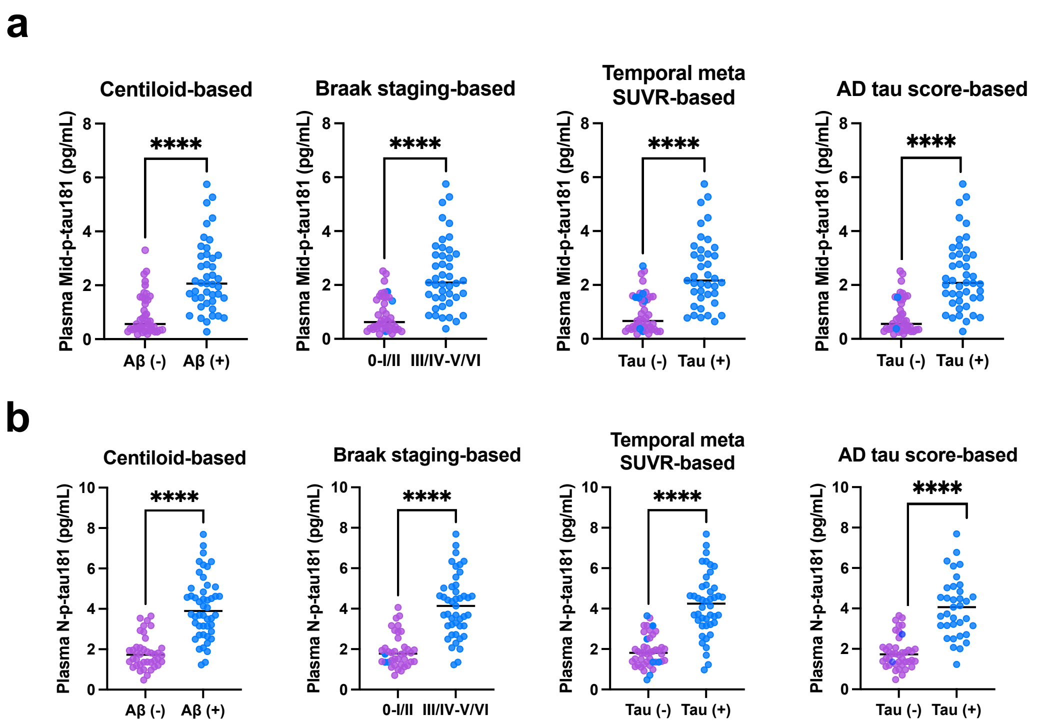

Fig. S7 Scatter plots of the mid-p-tau/ N-p-tau 181 showing its ability to discriminate between amyloid/tau PET status determined by semiquantitative approaches in the cognitively normal and AD continuum subjects. Scatterplots illustrating the relationship between mid-p-tau 181 (**a**) and N-p-tau181 (**b**) and levels and positive/negative amyloid/tau PET results as determined by semi-quantitative approaches. In the scatterplot, CN subjects are represented in purple, whereas AD continuum patients are depicted in blue. AD continuum was defined as MCI/AD. $P < 0.0001$ (****), as assessed by Mann–Whitney U test. Performance of mid-p-tau181/ N-p-tau181 in discriminating amyloid/tau PET status was determined using multiple semiquantitative approaches of evaluating brain amyloid/tau burden on PET in the CN and AD continuum subjects.

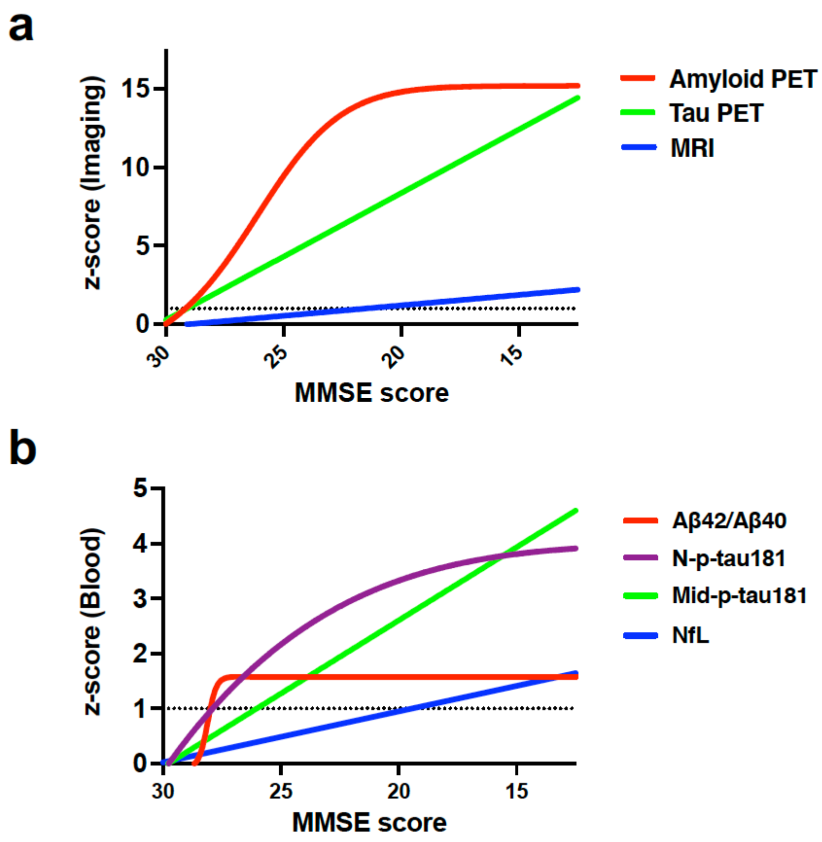

Fig. S8 Trajectories of the imaging and plasma A/T/N biomarkers along with the decline in the MMSE scores. Trajectories of the changes in imaging (**a**) and blood-based (**b**) A/T/N biomarkers with the decline in MMSE scores. The relationship between MMSE scores of the CN and AD continuum subjects and the z-scores of each biomarker is presented as a regression line that is either straight or sigmoidal, with the best fitting model being selected. The biomarkers were distinguished using red, green, and blue for both imaging and blood-based biomarkers, whereas N-p-tau181 was presented separately in purple. The dotted line indicates z-score = 1.

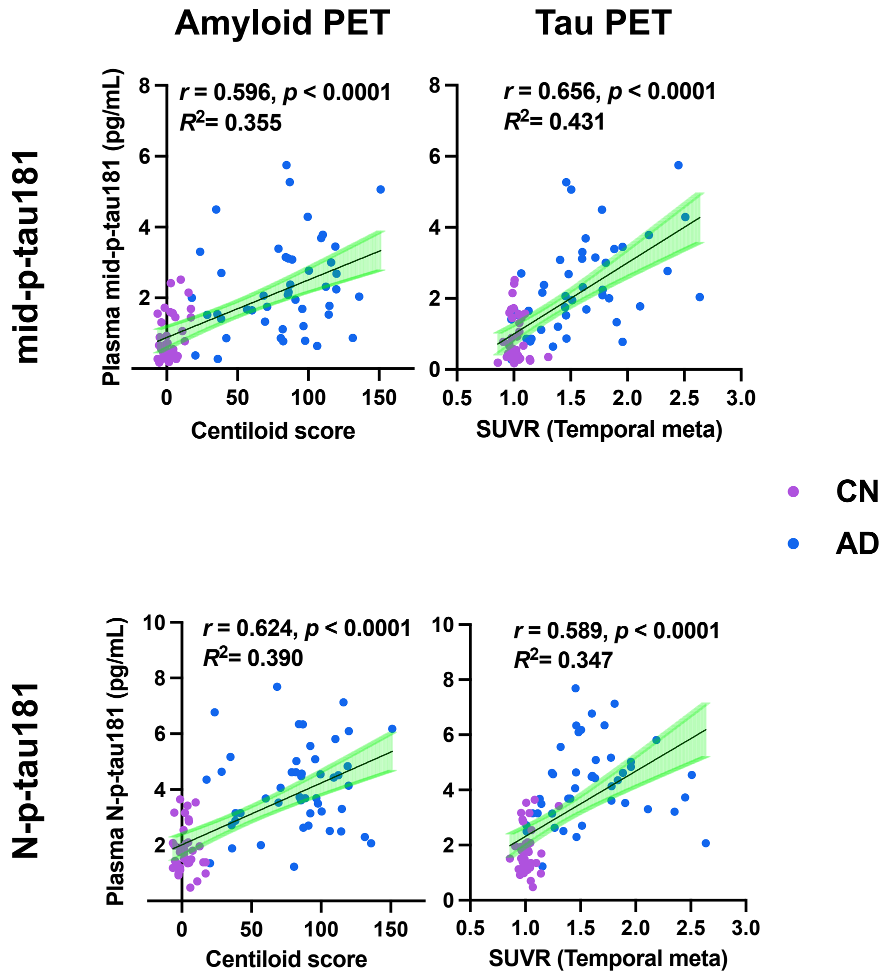

Fig. S9 Correlations between plasma p-tau levels assessed by each assay and amyloid/tau PET in the subjects with CN and AD continuum. All *r* values were estimated using Pearson's correlation analysis. In regression analyses, a linear model was selected in all cases.

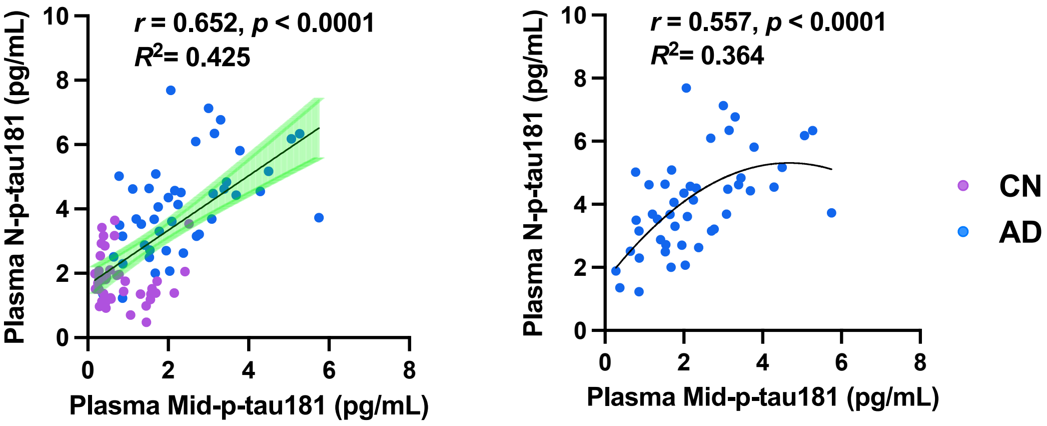

Fig. S10 Correlations between plasma mid-p-tau and N-p-tau levels in the subjects with AD continuum. Left panel shows the correlation between N-p-tau and Mid-p-tau levels for combined CN and AD groups. Right panel shows results for the AD group only.
