# Supplementary material for: A novel plasma p-tau181 assay as a specific biomarker of tau pathology in Alzheimer's disease
Source: Transl Neurodegener. 2024 Sep 3;13:44. doi: 10.1186/s40035-024-00439-4 (PMC11375937; doi:10.1186/s40035-024-00439-4)
Supplement: Supplementary file 2 — Additional file 2: Table S1. Intra-assay precision (n = 20). Table S2. Inter-assay precision for quality control samples (n = 25). Table S3. Recovery rate (%Recovery) for each plasma sample in the spike recovery tests. Table S4. An additional study of intra-assay reproducibility (%CV) by using the data on duplicated measurement of internal quality controls when the levels of plasma mid-p-tau181 were measured in the participants. Table S5. An additional study of inter-assay reproducibility (%CV) by using the data on repeated measurement of internal quality controls when the levels of plasma mid-p-tau181 were measured in the participants. Table S6. Demographic and blood biomarker data of the participants. Table S7. Differences in AIC values for regression analyses of p-tau levels and tau PET parameters. Table S8. Multiple linear regression analysis of p-tau levels with amyloid and tau PET metrics. Table S9. Amyloid/Tau PET status of cognitively normal individuals and AD continuum patients assessed by semi-quantitative approaches. Table S10. Performance of mid-p-tau 181and N-p-tau181assays in discriminating amyloid/tau PET status in the CN and AD continuum subjects. [file 40035_2024_439_MOESM2_ESM.pdf]

Additional file 2

Table S1. Intra-assay precision (n = 20).

	Sample 1: 1.56 pg/ml	Sample 2: 6.25 pg/ml	Sample 3: 25.0 pg/ml	Sample 4: 100 pg/ml
Mean AEB	0.0189	0.0580	0.224	0.872
SD	0.000817	0.000849	0.00656	0.00940
CV (%)	4.32	1.46	2.94	1.08

AEB, average enzyme per bead; SD, standard deviation; CV, coefficient of variation.

Table S2. Inter-assay precision for quality control samples (n = 25).

	Sample 1 (plasma)	Sample 2 (plasma)	Sample 3 (rec)	Sample 4 (rec)	Sample 5 (rec)
Mean AEB	0.00948	0.116	0.0592	0.128	0.882
SD	0.000738	0.00650	0.00545	0.00676	0.0526
CV (%)	7.78	5.60	9.19	5.30	5.96

AEB, average enzyme per bead; SD, standard deviation; CV, coefficient of variation.
plasma, plasma sample containing mid-p-tau; rec, recombinant p-tau protein.

Table S3. Recovery rate (%Recovery) for each plasma sample in the spike recovery tests.

	Treatment	Measured concentration (pg/ml)	CV (%)	Theoretical concentration of the spiked sample (pg/ml)	Recovery rate (%)
Sample 1	Neat (0 pg spike)	0.2675	14.9		
	0.15 pg spike	0.3500	7.94	0.4175	119.3
	0.3 pg spike	0.5130	12.2	0.5675	110.6
	0.6 pg spike	0.8649	7.73	0.8675	100.3
Sample 2	Neat (0 pg spike)	0.4516	6.91		
	0.15 pg spike	0.6842	3.63	0.6016	87.9
	0.3 pg spike	0.8909	5.82	0.7516	84.4
	0.6 pg spike	1.101	7.44	1.0516	95.6

CV, coefficient of variation.

Table S4. An additional study of intra-assay reproducibility (%CV) by using the data on duplicated measurement of internal quality controls when the levels of plasma mid-p-tau181 were measured in the participants

	Day 1	Day 2	Day 3	Day 4	Day 5
IQC 1 (recombinant)	4.86	2.27	0.03	0.92	4.38
IQC 2 (recombinant)	0.75	2.82	0.88	3.40	1.48
IQC 3 (plasma)	4.31	14.89	6.25	6.56	1.48
IQC 4 (plasma)	4.38	7.57	3.15	2.19	0.94

IQC, internal quality controls.

Table S5. An additional study of inter-assay reproducibility (%CV) by using the data on repeated measurement of internal quality controls when the levels of plasma mid-p-tau181 were measured in the participants

	IQC 1	IQC 2	IQC 3	IQC 4
	0.1172	0.8772	0.0139	0.0150
	0.1256	0.8679	0.0147	0.0141
	0.1205	0.8744	0.0158	0.0157
	0.1167	0.8403	0.0128	0.0141
	0.1341	0.9311	0.0126	0.0126
	0.1341	0.9429	0.0115	0.0120
	0.1214	0.7975	0.0130	0.0129
	0.1198	0.8368	0.0119	0.0125
	0.1230	0.8568	0.0127	0.0126
	0.1309	0.8391	0.0129	0.0124
Mean	0.1243	0.8664	0.0132	0.0134
SD	0.00657	0.04393	0.00130	0.00124
CV%	5.28	5.07	9.90	9.27

IQC, internal quality controls.

Table S6. Demographic and blood biomarker data of the participants.

	CN	AD	PSP	FTLD
Demographics				
Number	40	48	50	26
Age	66.0 (10.4)	69.3 (11.4)	71.2 (7.5)	65.0 (11.4)
Gender (male/female)	21/19	27/21	21/29	18/8
Years of schooling	14.8 (1.6)	13.9 (2.2)	13.6 (2.6)	14.0 (2.8)
MMSE	29.3 (1.0) †	21.9 (4.1) *	24.8 (5.8) *†	23.8 (6.1) *
FAB	16.7 (1.2) †	13.0 (3.0) *	12.0 (3.6) *	11.0 (4.8) *
CDR (0.5/1/2/3)	N/A	23/20/4/1	N/A	N/A
PSPRS	N/A	N/A	38.1 (18.2)	N/A
Blood biomarkers				

Aβ42/40	0.093 (0.020)†	0.067 (0.020)†	0.096 (0.048)†	0.089 (0.022)†
N-p-tau181 (pg/ml)	1.82 (0.79)†	4.07 (1.52)*	2.27 (1.06)†	2.25 (1.25)†
mid-p-tau181 (pg/ml)	0.83 (0.65)†	2.30 (1.31)*	1.56 (0.91)*	0.96 (0.63)†
NfL (pg/ml)	19.5 (12.5)†‡	33.2 (21.3)*‡	58.3 (39.7)*†	51.8 (39.3)*

This table summarizes the demographic information of the participants. All participants (*n* = 164) underwent neuropsychological assessment, simultaneous amyloid and tau PET imaging, and blood sampling on the day of the PET examination. CN individuals exhibiting negative results on amyloid and tau PET imaging were designated into the CN cohort. Patients with MCI and AD who had positive amyloid PET findings were categorized into the AD continuum group (dubbed AD group). Furthermore, subjects with PSP and other FTLD who had negative amyloid PET findings were classified into the PSP and FTLD cohorts, respectively. No significant differences in age, years of schooling, and gender were observed among the groups. Notably, 90% of the AD group comprised subjects with early-stage AD who had a CDR score of 0.5 or 1 accounted. CN, cognitively normal; AD, Alzheimer's disease; PSP, progressive supranuclear palsy; FTLD, frontotemporal degeneration; MMSE, Mini-Mental State Examination; FAB, Frontal Assessment Battery; CDR, Clinical Dementia Rating scale; PSPRS, progressive supranuclear palsy rating scale; N/A, not applicable; Aβ, amyloid beta; N-p-tau181, phosphorylated tau181 measured using the commercial kit directed to the C-terminally truncated N-terminal fragment of p-tau (Simoa pTau-181 V2.1 Assay, Quanterix); mid-p-tau181, phosphorylated tau181 measured using the originally developed immunoassay directed to both the N- and C-terminally truncated p-tau181 fragments; NfL, neurofilament light chain.

Values are presented as mean ± standard deviation.

*, Significant difference between CN, †, between AD, ‡, between PSP, $P < 0.05$ (corrected by Dunn's multiple comparisons).

Table S7. Differences in AIC values for regression analyses of p-tau levels and tau PET parameters

		Tau PET SUVRs			
	AD tau score	Temporal meta	Braak I/II	Braak III/IV	Braak V/VI
Mid-p-tau181	1.24	1.12	-2.42	2.11	1.98
N-p-tau181	-3.12	-18.15	0.047	-12.57	-7.99

The values represent the differences in AIC between linear and nonlinear regression analyses. Positive values indicate that the linear regression model is preferred, while negative values indicate that the nonlinear regression model is more suitable.

Table S8. Multiple linear regression analysis of p-tau levels with amyloid and tau PET metrics

	Beta coefficient	Standard error	Adjusted R^2	*P*-value
mid-p-tau181				
Amyloid PET (Centiloid)	0.258	0.115	0.450	0.056
Tau PET (Temporal meta-ROI)	0.472	0.115		<0.0001
N-p-tau181				
Amyloid PET (Centiloid)	0.399	0.118	0.420	0.002
Tau PET (Temporal meta-ROI)	0.311	0.118		0.020

Table S9. Amyloid/Tau PET status of cognitively normal individuals and AD continuum patients assessed by semi-quantitative approaches

	CN		AD	
Quantitative approach	Amyloid/Tau (-)	Amyloid/Tau (+)	Amyloid/Tau (-)	Amyloid/Tau (+)
Centiloid (Amyloid)	40	0	3	45
Braak staging (Tau)	35	5	3	45
Temporal meta-ROI SUVR (Tau)	37	3	7	41
AD tau score (Tau)	40	0	2	35

The cut-off values of Centiloid, Temporal meta-ROI SUVR and AD tau score were 24, 1.105 and 0.1986, respectively. AD tau scores were exclusively computed for cases that underwent imaging with the mCT PET scanner.

Table S10. Performance of mid-p-tau 181and N-p-tau181assays in discriminating amyloid/tau PET status in the CN and AD continuum subjects

	Sensitivity (%)	Specificity (%)	Cutoff value (pg/ml)	AUC
mid-p-tau181				
Centiloid	95.4	60.5	0.749	0.845
Braak staging	62.8	92.5	1.76	0.859
Temporal meta SUVR	71.8	88.6	1.69	0.855
AD tau score	70.5	88.1	1.62	0.870
N-p-tau181				
Centiloid	91.1	76.7	2.20	0.890
Braak staging	91.2	76.3	2.21	0.903
Temporal meta SUVR	77.3	95.5	3.19	0.910
AD tau score	91.4	78.6	2.20	0.912

All parameters were estimated from receiver operating characteristic curve analyses. We set each cutoff value according to Youden index obtained in the respective receiver operating characteristic analyses.

SUVR, standardized uptake value ratio; AUC, area under the receiver operating characteristic curve.
